# Relapse of imported *Plasmodium vivax* malaria is related to primaquine dose: a retrospective study

**DOI:** 10.1186/1475-2875-11-214

**Published:** 2012-06-22

**Authors:** Nicola Townell, David Looke, David McDougall, James S McCarthy

**Affiliations:** 1 Infection Management Services, Princess Alexandra Hospital, Brisbane, QLD, 4102, Australia; 2 Southern Clinical School, Faculty of Health Sciences, University of Queensland, Queensland, Australia; 3 Queensland Institute of Medical Research, University of Queensland, Queensland, Australia; 4 Department of Infectious Diseases Royal Brisbane and Women’s Hospital, Herston, QLD, 4029, Australia

**Keywords:** *Plasmodium vivax*, Relapse, Primaquine, Imported, Epidemiology, Australia, Oceania

## Abstract

**Background:**

Relapsing *Plasmodium vivax* infection results in significant morbidity for the individual and is a key factor in transmission. Primaquine remains the only licensed drug for prevention of relapse. To minimize relapse rates, treatment guidelines have recently been revised to recommend an increased primaquine dose, aiming to achieve a cumulative dose of ≥6 mg/kg, i.e. ≥420 mg in a 70 kg patient. The aims of this study were to characterize the epidemiology of *P*. *vivax* infection imported into Queensland Australia, to determine the rates of relapse, to investigate the use of primaquine therapy, and its efficacy in the prevention of relapse.

**Methods:**

A retrospective study was undertaken of laboratory confirmed *P. vivax* infection presenting to the two major tertiary hospitals in Queensland, Australia between January 1999 and January 2011.

Primaquine dosing was classified as no dose, low dose (<420 mg), high dose (≥420 mg), or unknown. The dose of primaquine prescribed to patients who subsequently relapsed that prescribed to patients who did not relapse.

**Results:**

Twenty relapses occurred following 151 primary episodes of *P*. *vivax* infection (13.2%). Relapses were confirmed among 3/21 (14.2%), 9/50 (18.0%), 1/54 (1.9%) and 7/18 (38.9%) of patients administered no dose, low dose, high dose and unknown primaquine dose respectively. High dose primaquine therapy was associated with a significantly lower rate of relapse compared to patients who were prescribed low dose therapy (OR 11.6, 95% CI 1.5-519, p = 0.005).

**Conclusions:**

Relapse of *P. vivax* infection is more likely in patients who received low dose primaquine therapy. This study supports the recommendations that high dose primaquine therapy is necessary to minimize relapse of *P. vivax malaria.*

## Background

Although Australia was declared malaria free in 1981, cases of malaria continue to be imported into the country, with *P. vivax* being the most common causative species [1,2]. The state of Queensland reports the greatest numbers, at a rate nearly double that of the Australian average [2]. Most of these infections are acquired in Oceania, with Papua New Guinea (PNG) being the source of over two-thirds of malaria imported into Queensland [1,3,4]. Of note, over the past decade there has been a gradual reduction in notifications of malaria into Australia, with laboratory diagnoses in the state of Queensland falling from 504 in 2000 to 146 in 2011 (McCarthy, unpublished).

Primaquine, an 8-aminoquinoline, has been in widespread clinical use for over 60 years, and remains the only treatment option for eradication of liver hypnozoites and, therefore the prevention of relapses [5,6]. The mechanism of action of primaquine is poorly understood [6,7]. It is widely available, affordable and is generally well-tolerated [8]. Gastrointestinal symptoms, while common can be minimized by taking the medication with food or by splitting doses [9]. Primaquine therapy can result in severe acute haemolysis in patients with glucose-6-phosphate dehydrogenase (G6PD) deficiency. It is, therefore, contraindicated in patients with G6PD deficiency and pregnancy (due to the unknown G6PD status of the foetus) [9]. G6PD testing is essential, but imposes significant costs and logistical problems, especially in resource poor settings, where it inhibits widespread use.

The efficacy of primaquine therapy in preventing relapses is difficult to define, with studies confounded by uncertain compliance, recrudescence and re-infection [6,10]. To minimize these confounding effects, assessment of primaquine efficacy can be most readily studied in travellers returning to developed non-malarious regions. There is, however, a paucity of studies from these regions that have specifically focused on primaquine use and dosing.

Traditionally, the recommended dose of primaquine has been 15 mg/day for 2 weeks. The rationale for the licensing of this dose was based predominantly on safety – 15 mg/day could be administered to African-American soldiers, returning from the Korean war, without concerns of life-threatening haemolysis [7]. The efficacy of primaquine therapy for clearance of hepatic stages of *P. vivax* is considered to vary according to the geographical area of acquisition [10]. Although treatment failures have been documented from all geographic regions, generally poorer responses have been observed among patients acquiring their malaria from SE Asia and Oceania [5,10], with the so-called Chesson strain being documented to be relatively resistant to primaquine [5]. Over the past decade, there has been increasing evidence that higher cumulative doses of primaquine are required to reduce treatment failures [11-13], and as a consequence the recommended cumulative dose of primaquine has been recently revised upwards to 6 mg/kg (i.e. 30 mg/day for 14 days in a 70 kg patient) [5,7]. This high dose primaquine regimen is now recommended as standard of care in an increasing number of guidelines [14-16]. Since 2010, Australian guidelines have been recommending ≥ 6 mg/kg (i.e. 30 mg for 14 days in a ≤70 kg patient) [16]. The latest WHO treatment guidelines currently recommend high dose therapy only for patients who acquire their infection from SE Asia and Oceania [17].

The aims of this study were to characterize the epidemiology of *P*. *vivax* infection currently imported into Queensland Australia, to determine the rates of relapse, to investigate the use of primaquine therapy and its efficacy in the prevention of relapse.

## Methods

A retrospective review of *P*. *vivax* malaria cases was performed in the two major adult tertiary hospitals in south-east Queensland (Princess Alexandra Hospital and Royal Brisbane and Women’s Hospital). Cases of *P. vivax* infection were identified by computerized search of the state-wide pathology records from January 1999 to January 2011. Cases were defined as *P. vivax* malaria based either on blood film or a PCR test.

To optimize ascertainment of cases and relapses, collateral information was collected from the Queensland Public Health Malaria Register, Sullivan and Nicolaides Pathology (one of the two major private pathology laboratories in Queensland) and from the Queensland Malarial Reference Laboratory (QMRL). Since 1999, all public hospitals and both major private laboratories in Queensland have routinely referred all positive blood films to QMRL for confirmatory slide verification. All available medical charts were reviewed, data were de-identified and entered into a Microsoft Excel spreadsheet (v2003). Data collected included epidemiological data, travel history, severity of illness, diagnosis, treatment, primaquine therapy and outcomes. Chemoprophylaxis regimens were considered appropriate if concordant with Australian guidelines [16], which are closely aligned to other international guidelines.

A relapse was defined as a subsequent infection with no return travel to a malaria endemic country in the interval between first and subsequent episode, or *P. vivax* infection after receiving primaquine therapy as presumptive anti-relapse therapy (PART, otherwise known as terminal prophylaxis). If a patient had received chloroquine therapy for treatment of *P. vivax* infection, incident malaria within 35 days could either be due to relapse or recrudescence or both [18]. The authors were unable to classify the subsequent infection in these patients. For the purpose of this study, in order to determine the adequacy of primaquine at preventing relapses, they were defined as having recrudescent parasitaemia and excluded from further analysis. Re-infection was defined as a second infection that occurred following subsequent travel to an endemic country.

As patients’ weights were not universally recorded, a weight of 70 kg was empirically applied to all patients. For the purpose of the study, primaquine dosing was defined as no dose, low dose (<420 mg; assuming a weight of 70 kg), high dose (≥420 mg; equivalent to 6 mg/kg in a 70 kg patient) or unknown dose. Patients were censored with no further statistical analysis after their first relapse to prevent confounding by the cumulative effect of repeated primaquine dosing.

Statistical analysis was performed using Microsoft Excel (v2003) and Stata V11. Categorical data was analysed using both Fischer’s exact tests with two tailed p-values and calculation of Odds ratio (OR) with 95% confidence intervals (CI), and is logistic regression models where appropriate. Nonparametric continuous data was analysed using Wilcoxon rank-sum test. P-value of less than 0.05 was deemed to be statistically significant. Ethics approval was gained from the responsible ethics committees at both institutions (HREC/10/QPAH/338,HREC/10/QRBW/361).

## Results

**Figure 1:**
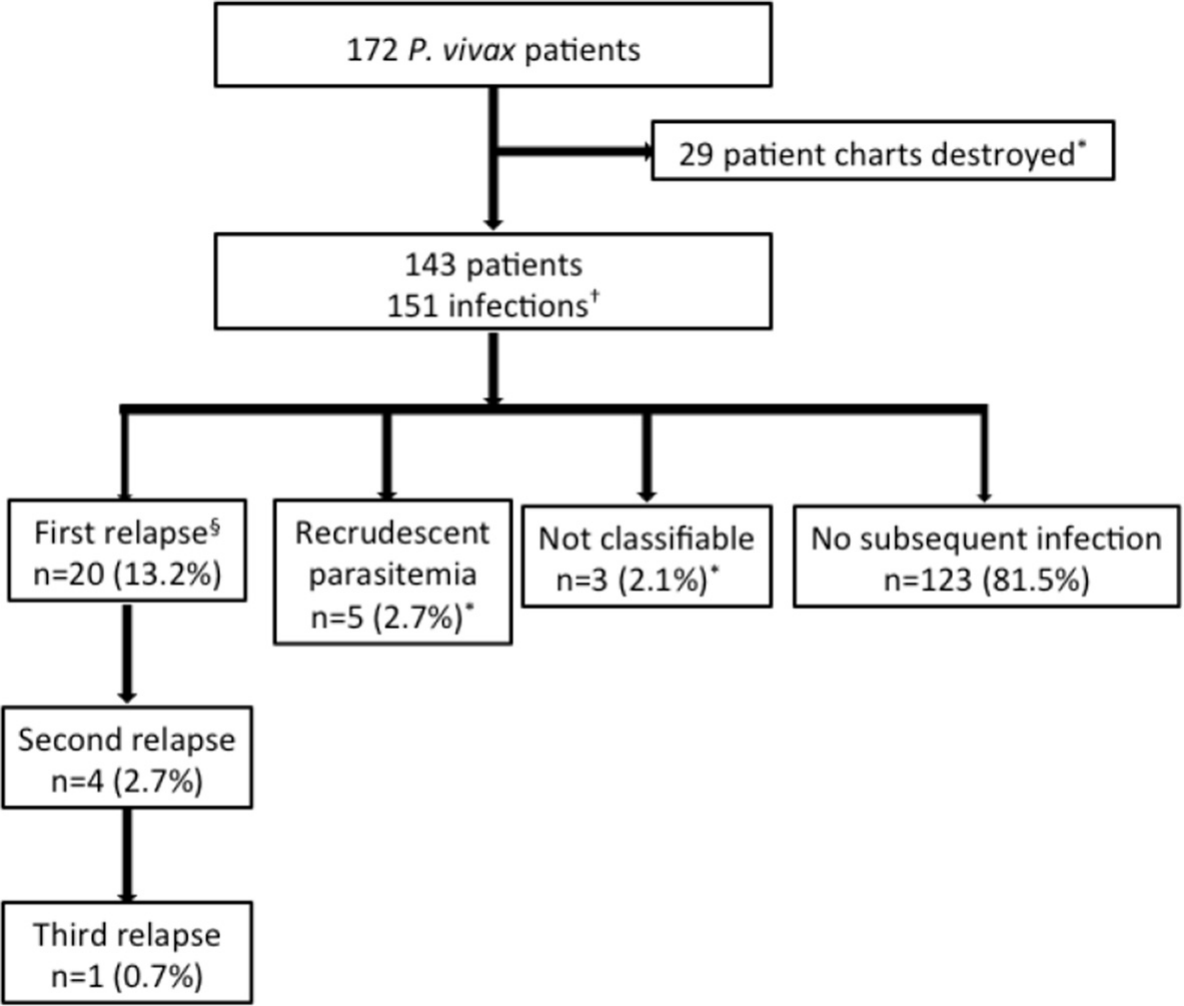
**Patient flow diagram showing subsequent infection outcomes. **Percentages based on number of primary infections. † includes 8 subjects with reinfections. · excluded from analysis. § includes 2 patients who received PART.

A patient flow diagram is shown in Figure 1. *Plasmodium vivax* infection was identified in 172 patients between January 1999 and January 2011. The medical records of 29 patients had been destroyed, and so these cases were excluded from the analysis. All remaining patients (n = 143) were included in the study. A total of 184 *P. vivax* infections were evaluated, of which 151 were episodes of primary *P. vivax* infection. Eight (5.3%) second infections occurred following a subsequent visit to a malaria endemic region, and were classified as reinfections. Following chloroquine therapy, five (3.3%) second infections occurred within 35 days of the initial diagnosis (days 20, 20, 22, 23, 27). All five patients had travelled to PNG, had received chloroquine therapy, and had a recurrent *P. vivax* diagnosis confirmed. As it was not possible to determine whe-ther their recurrent infection represented recrudescent chloroquine-resistant *P. vivax* infection or relapse, for the purpose of this study their recurrent infections were classified as recrudescent parasitaemia. Three patients had a second infection for which no further clinical information was available; these infections could not be classified and were excluded from further analysis. Twenty (13.2%) relapses were recorded, including two patients who had received PART. Four (2.7%) patients subsequently had a second relapse and one (0.7%) patient had a third relapse. Mixed infections occurred in 7.3% (n = 11) of cases, with ten cases of *P. falciparum* infection and one case of *Plasmodium malariae* infection.

Patient demographics are shown in Table 1. Over three quarters of *P. vivax* infections were imported from Oceania (n = 115, 76.2%). The most commonly visited countries were PNG (n = 91, 60.3%), followed by the Solomon Islands (n = 15, 9.9%), India (n = 14, 9.3%), Indonesia (n = 10, 6.6%) and Vanuatu (n = 8, 5.3%). The main reasons for travel were recorded as business (n =47, 31.1%), tourism (n = 30, 19.9%), and visiting friends or relatives (VFR) (n = 28, 18.5%). Less than half of the patients were recorded as having taken chemoprophylaxis (n = 64, 42.4%). For those who received chemoprophylaxis, doxycycline was the preferred agent (n = 39, 60.9%). The chemoprophylactic regimen was judged inappropriate in 6 (9.4%) cases; all entailed the prescription of chloroquine in patients travelling to Oceania.

**Table 1:** Patient characteristics

		N (%)*	**Median**	**IQR (range)**
Age – years			32	23-47 (13–76)
Duration of trip – months (n = 66)			1	0.8-3 (0.1-19)
Weight – kg (n = 43)			72	66-89 (52–104)
Male Gender		105 (69.5)		
Reason for travel	Business	47 (31.1)		
	Tourist	30 (19.9)		
	VFR^	28 (18.5)		
	Migrant	18 (11.9)		
	Foreign visitors	10 (6.6)		
	Not Stated	18 (11.9)		
Geographic region visited	Oceania	115 (76.2%)		
	Indian subcontinent	14 (9.3%)		
	SE Asia	11 (7.3%)		
	Africa	6 (4.0%)		
	S. America	2 (1.3%)		
	Multiple	2 (1.3%)		
	Unknown	1 (0.7%)		
Chemoprophylaxis	Yes	64 (42.4)		
	No	77 (51.0)		
	Not recorded	10 (6.6)		
Chemoprophylaxis agent (n = 64)	Doxycycline	39 (60.9)		
	Chloroquine	6 (9.4)		
	Mefloquine	5 (7.8)		
	Atovaquone/proguanil	2 (3.1)		
	Sulphadoxine/pyrimethamine	1 (1.6)		
	Combination of agents	2 (3.1)		
	Agent not known	9 (14.0)		
Mixed infection	*P. falciparum*	10 (6.6)		
	*P. malariae*	1 (0.6)		
Treatment (n = 179)	Chloroquine	115 (64.2)		
	Artemether/lumefantrine	12 (6.7)		
	Quinine+doxycycline	7 (3.9)		
	Mefloquine	5 (2.8)		
	Artesunate	1 (0.6)		
	Atovaquone/proguanil	1 (0.6)		
	Combination	11 (6.1)		
	Change in regimen	18 (10.5)		
	Unknown	9 (5.0)		

*Percentages based on number of primary P. Vivax infections, unless stated otherwise.

^VFR – visiting friends and relatives.

The median time from arrival into Australia until diagnosis of infection was one month, with a range of 0 days to 14 months (IQR 0.4-2.6 months). *P. Vivax* malaria was a benign infection in the majority of the patients, with no recorded deaths. Two (n = 2/179, 1.1%) infections were defined as severe [17]–one patient had severe anaemia and required a blood transfusion, while one patient had mixed infection with *P. falciparum* and required an ICU admission due to multiple convulsions and hypernatremia*.*

Over half of all infections (n = 98/179, 54.8%) were initially managed as an inpatient, with a median length of stay of 1 day (IQR 1–3). Chloroquine was the preferred initial treatment agent (n = 115, 64.2%). Artemisinin combination treatment regimens were used in 12 (6.7%) infections, and were used with increasing frequency from 2008 onwards. A change of treatment regimen occurred in 18 (10.1%) cases. Reasons reported for change in therapy included poor clinical response, or clinician preference. Clinical failure of chloroquine therapy was noted in 6/115 (5.2%) infections – all patients acquired malaria from PNG or nearby islands, and had prolonged fever (≥three days after commencement of therapy) necessitating change in therapy which was followed by subsequent clinical improvement. No severe adverse effects of therapy were noted.

The median time between infection and relapse was 78 days (IQR 60–119, range 27–399). Although not statistically significant, there was a trend for increased incidence of relapse in patients returning from Oceania (n = 18/107, 16.8% vs. n = 2/36 5.6% rest of the world, OR 3.4 CI 0.75-31.9, p = 0.09).

Various primaquine regimens were prescribed (od/bd/tds/qid, 7.5 mg/15 mg/22.5 mg/30 mg, 1 week/2 weeks/3 weeks). Over 75% (n = 114, 79.7%) of patients received primaquine therapy after their initial infection. Primaquine was not given to 21 subjects for the following reasons: planned return to an endemic country (n = 13, 61.9%), failure to attend clinic (n = 4, 19.0%), pregnancy (n = 1, 4.8%), allergy (n = 1, 4.8%), mistake – film thought to be *P. falciparum* (n = 1, 4.8%) and unknown (n = 1, 4.8%). Primaquine therapy was well tolerated, with no major adverse events or drug discontinuations recorded. G6PD status was not assessed in 9 (7.9%) patients despite them receiving primaquine. No cases of G6PD deficiency were identified.

Primaquine dosing and outcomes are shown in Table 2. The dose of primaquine prescribed was not documented in 18 (12.6%) infections. The median cumulative prescribed dose of primaquine was 315 mg (IQR 210-420 mg). The cumulative dose of primaquine therapy in patients who relapsed was lower compared to the dose received by patients who did not relapse (median 210 mg, IQR 210-315 mg vs. median 315 mg, IQR 210–420, p = 0.01). Patients who received low dose primaquine therapy were significantly more likely to relapse compared to patients who received high dose primaquine therapy (OR 11.6, 95% CI 1.5-519, p = 0.005).

**Table 2:** Primaquine dose

				**Relapsed**		**Infection without**	
		**Overall (n = 143)**		**Infection (n = 20)**		**relapse (n = 123)**	
Primaquine		N	(%)	N	(%)	N	(%)
	Yes	114	79.7	16	80.0	98	79.7
	Nil	21	14.7	3	15.0	18	14.6
	Unknown	8	5.6	1	5.0	7	5.7
Primaquine dose							
	Nil	21	14.7	3	15.0	18	14.6
	Low (<420 mg)	50	35.0	9†	45.0	41†	33.3
	High (≥420 mg)	54	37.8	1†	5.0	53†	43.1
	Unknown	18	12.6	7	35.0	11	8.9
Dosage (mg)		median	(IQR)	median	(IQR)	median	(IQR)
		315	(210–420)	210	(210–315)	315	(210–420)

†(OR 11.6, 95% CI 1.5-519, p = 0.005).

## Discussion

Accurate ascertainment of relapse rates in *P. vivax* malaria is well recognized to be difficult, with studies often compromised by under-reporting and short follow-up periods. In this study, the computerized state-wide pathology services, and the compulsory reporting of all cases to the Queensland Malarial Reference Laboratory made it possible to confidently identify subsequent *P. vivax* infections occurring within the State. Incomplete ascertainment of relapses remains possible, as a patient may have relapsed whilst interstate or abroad. Other limitations of the study include its retrospective design, resulting in potential loss of clinical information, non-standardized treatment regimens, and no active follow up with consequent unknown adherence to treatment. Despite these limitations, the relapse rate of 18.0% following low dose primaquine therapy is similar to what has been reported in other studies of imported *P. vivax* malaria [11,19-25]. These data are also in agreement with other published studies that have reported a significantly lower frequency of relapse with increased primaquine dosing [6,9,11,13,17], and support the hypothesis that relapse rates are low (<5%) if high dose primaquine therapy is prescribed. Unfortunately, the implementation of evidence-based dose regimens of primaquine in endemic countries remain hampered by widespread use of, low dose, short course therapies for which a paucity of efficacy data are available [13].

The data support the recommendation that, in order to minimize treatment failure, it is important to prescribe the target dose based on patient weight [11]. Although weights were not available for all patients, our estimated weight of 70 kg was similar to the median weight of 72 kg of patients whose weight was recorded (IQ R66-89, n = 43). Thus, 420 mg of primaquine, which is equivalent to a dose of 6 mg/kg, would be considered appropriate as a high dose regimen for half of the study population.

Although compliance could not be assessed in this study, the importance of compliance in reducing relapses is well documented [8]. Compliance may be improved by providing patients with education regarding the need for primaquine as well as regular review. It is also possible that compliance may be improved by using a high dose abbreviated regimen, such as 30 mg twice daily for 1 week, in patients who are not G6PD deficient [5].

Further studies are required to determine if high dose therapy is the optimal dose of primaquine. Ideally, a randomized controlled trial would be performed, but this is unlikely to be undertaken due to logistic constraints. Thus, retrospective studies, such as this study, may be an important strategy to gather further information.

Only 3 patients of 21 (14.3%) who were not prescribed primaquine relapsed. As most patients acquired their infection from Oceania, this relapse rate is lower than what may have been expected. It is, however, not significantly different to the frequency of recurrence in this study among patients who were prescribed low dose primaquine therapy (3/21 [14.3%] *vs* 9/50 [18%]). Further, most of the patients who did not receive primaquine were planning to return to an endemic country soon, or failed to attend clinic (n = 17, 80.1%). It is, therefore, possible that incomplete ascertainment of relapses in this group may have resulted in a lower reported frequency of recurrence.

There are a few published case reports of relapses despite patients being prescribed 420 mg of primaquine [12,26]. These cases are clustered around Southeast Asia and Oceania – two cases from Indonesia and each respectively from Vanuatu and the Solomon Islands. In none of these three cases nor in the single case reported in this study was weight-based dosing and/or compliance reported. This case, as with the three other cases described, does not satisfactorily document either weight-based dosing and/or compliance. Subsequent primaquine treatment of these patients varied - one patient refused further primaquine after his 3^rd^ relapse, the patient in this study received 315 mg, two patients received 840 mg (60 mg/day for 14 days), all without complication or subsequent relapse. The most appropriate management approach for patients who fail high dose therapy remains unclear. Further studies are required to determine the ongoing adequacy of high dose primaquine therapy and the potential development of primaquine resistance. If treatment failures are subsequently identified, such cases need to be further evaluated in respect to causes, treatment and prevention.

The majority of *P*. *vivax* infections in Australia are imported from Oceania, especially PNG. Although not statistically significant, our study supports the previously reported increased risk of relapse if *P. vivax* is acquired in Oceania [19,25,27].

## Conclusion

This study shows that high doses of primaquine (≥420 mg) are associated with lower rates of relapse of *P. vivax* infection, and supports current treatment guidelines recommending ≥6 mg/kg (i.e. 30 mg/kg for 14 days in a ≤70 kg patient) total dose.

## Competing interest

The authors declare no conflicts of interests.

## Authors’ contribution

Conceived study: NT, DL, JMC; collected data: NT; analysed data: all authors; drafted manuscript: NT; reviewed and edited manuscript all authors. All authors read and approved the final manuscript.
